# The coupling coordination between rural public services and rural tourism and its causative factors: The case study of southwestern China

**DOI:** 10.1371/journal.pone.0290392

**Published:** 2023-08-24

**Authors:** Shiqiao Fang, Kaihang Ou, Jing Xiong, Rongmei Teng, Lifang Han, Xufan Zhou, Hongyu Ma

**Affiliations:** 1 School of Tourism and Culture, Nanning Normal University, Nanning, China; 2 Guangxi Tourism Research Institution, Guilin, China; 3 School of Geographical Science and Planning, Nanning Normal University, Nanning, China; 4 School of Sports and Health, Nanning Normal University, Nanning, China; Shanghai Dianji University, CHINA

## Abstract

Rural public services and rural tourism are interdependent, and their coordinated development is crucial for promoting rural revitalization and overall growth in China. So far, the existing studies mainly focus on the mutual influence, mutual promotion, and coordination paths of rural public services and rural tourism but fail to conduct an empirical analysis on the coupling coordination of rural public services and rural tourism or summarize the spatial and temporal differences of the coupling coordination. Therefore, we adopt an evaluation index system for rural public services and rural tourism. To measure the development level and the coupling coordination degree of rural public services and rural tourism in southwestern China from 2012 to 2019, we used a comprehensive evaluation model and a coupling coordination degree model. Additionally, geographic detectors were utilised to detect the causative factors of their coupling coordination development. Based on the analysis of research results, we made the following observations. In southwestern China, the comprehensive development of rural public services and rural tourism indicated an upward trend. An additional interactive coupling relationship between the two systems is observed, and its coupling coordination degree increases, with the increment varying from slow to rapid. The type of coupling coordination changes from rural tourism lagging type to rural public service lagging type, and there are spatial differences in the degree of coupling coordination between the two. The coupling coordination development of the two systems is affected by multiple causative forces, such as economic, industrial, resource attraction, and service guarantee forces, and some differences distinguish the driving strengths of both single and interaction factors. The main contribution of this article is to reveal the coupling and coordination relationship between rural public services and rural tourism, to explore the driving factors affecting the degree of coupling and coordination between them, and to make relevant policy recommendations.

## 1. Introduction

Rural public services play a dual role in rural areas, offering essential services to local residents while also providing comprehensive public services and commercial supporting facilities for tourists [1]. This synergy enhances the competitive advantages of rural tourism, making it more appealing and attractive. Over the years, rural tourism in China has experienced significant growth, expanding its scale since the beginning of the 21st century [2]. In 2019, China’s rural tourism had 3.009 billion visitors, with an average annual growth rate of more than 10% in the number of visitors. Rural tourism revenue exceeded 1.81 trillion yuan [3], and rural tourism has gradually developed into a strategic pillar industry for rural revitalization. Despite the rapid acceleration and expansion of rural tourism, the development of public services has struggled to keep pace with the increasing demand and supply pressure brought about by the growing number of tourists and their evolving needs [4]. Consequently, rural tourism has placed higher demands on public service facilities, necessitating a focus on enhancing and expanding these services to meet the requirements of the burgeoning tourism industry. Persistent imbalances in the supply of public services in rural China have arisen due to various factors, including the natural environment, socio-economic factors, and issues within the social sector. These challenges have contributed to disparities in the availability and quality of public services across different rural regions in the country, i.e., inadequate overall supply, low service levels, and limited utilization rates [5], which to a certain extent do not match the needs of the growing development of rural tourism, transforming it to a short-board that restricts the high-quality development of rural tourism [6]. This disparity is particularly evident in economically disadvantaged areas with limited access to natural resources [7, 8]. Therefore, speeding up to make up the shortfall in rural public services is an inevitable choice to promote rural revitalization and the high-quality development of rural tourism. Addressing the contradiction between the pressing demand for high-quality development of rural tourism and the relative inadequacy of public service facilities has become a shared focal point within the industry and the academic sector. Both contributors are actively seeking solutions to bridge this gap and promote sustainable and inclusive growth in rural tourism while simultaneously improving and modernizing public service infrastructure to meet the evolving needs of the market [9]. Nowadays, rural tourism and rural public services have formed a collaborative relationship to foster mutual development. As rural tourism thrives, it contributes positively to the growth of rural public services, while the existence of robust rural public service facilities serves as foundational support for the advancement of rural tourism. This beneficial interaction between the two sectors encourages coordination and facilitates mutual feedback, fostering a shared direction of development that is mutually reinforcing. The coupling of coordination and development between the two is important for promoting high-quality development and rural revitalization on both sides. However, it is not clear how they interact and achieve virtuous and mutual growth. Following previous, we can observe the influence they have on each other, as well as the formation of a complex relationship between the two [10–12], but it is still unclear how they interact and to what extent their actions are coordinated. Previous studies have mostly focused on the national [13], provincial [14], and economic belt [15], while little attention has been paid to southwest China as a region with relatively backward economic and social development. Therefore, the coupling relationship between rural public services and rural tourism needs to be studied further using southwestern China as a case study. The primary aim of this research is to guide rural society and economy towards achieving sustainable development more effectively. Additionally, the study seeks to expand the existing perspectives in sustainable development research [16, 17], particularly in the context of rural areas. By doing so, the research endeavors to offer valuable insights and solutions to foster long-term and balanced growth in rural regions.

In this context, this paper attempts to address the following scientific questions, using Southwest China as an example: To What is the degree of coordinated development of rural public services and rural tourism? What are the evolutionary characteristics of the coordinated development of the two systems? What are the causative factors that affect their coordinated development? These questions must be addressed, to make the relationship between rural public services and rural tourism more optimal.

## 2. Literature review

The study of rural public services and rural tourism has become a main point of discussion and has received increasing attention from various academic circles. Relevant research up to this day has mainly focused on the impact of rural public services on rural tourism, the impact of rural tourism on rural public services, and the associations between rural public services and rural tourism.

### 2.1 The impact of rural public services on rural tourism

Rural public services provide a solid foundation for rural tourism development. In rural areas, poor transportation planning can hinder tourists from accessing tourist attractions due to their spatial dispersion [18]. Therefore, infrastructure is an important determinant of a destination’s attractiveness and can impact its tourist development [19]. It can also effectively enhance value creation from tourism in the region [20]. Besides, good local basic education provides knowledge and support for rural tourism.

A low local education level can lead to a lack of profound understanding of rural tourism and basic tourism service skills among local practitioners. This, in turn, can have a significant impact on the development of rural tourism in the area [21]. Moreover, rural public environmental protection services have a positive impact on rural tourism development [22], and rural ecological environment improvement is the primary focus in the promotion and enhancement of rural tourism [23]. Similarly, quality health services can attract citizens who are concerned about their health and well-being as well as patients seeking quality services; they can, therefore, promote the tourism market [24]. The role of rural public cultural services extends beyond providing cultural and social education functions to also serving as rural tourism destinations and attractions. For instance, facilities like libraries [25] and museums [26] play a significant role in promoting the development of local rural tourism. In addition, frequent enhancement of local visibility through the organization of cultural activities generates a branding effect for tourism in the region [27]. Lastly, social security services play a crucial role in safeguarding farmers’ income against market fluctuations and mitigating the risks associated with their involvement in rural tourism [21].

### 2.2 The impact of rural tourism on rural public services

Rural tourism has an impact on rural public services by positively influencing rural infrastructure development and rural public culture and by promoting rural community development [28]. In addition, rural tourism significantly affects local education [29], and rural tourism influences the progress of basic education by encouraging the development of rural societies [30]. Similarly, rural tourism, as a new form of poverty alleviation in developing countries, can bring considerable economic benefits and improve the overall social security level of residents in rural areas [31].

Moreover, the tourist festivals held in some rural areas can significantly contribute to the enhancement of local social and cultural benefits [32] and lead to improved public cultural services. In this regard, rural tourism not only enables tourists to reconnect with the nature and society of the destination but also plays a role in environmental protection [33] and fosters the development of public health [34]. However, some scholars argue that developing countries are still sceptic about the economic development and environmental protection tourism encourages and that tourism development, while helping many people escape poverty, has also led to increased pollution and water scarcity [35], increasing the pressure for local public services. Some studies have also found that rural tourism development can cause a decline in the quality of local public health care services and mitigate public health care provision [36].

### 2.3 The interactions between rural public services and rural tourism

Tourism provides residents with opportunities to improve public services and enhance personalized services for tourists [37]. In turn, public service facilities promote tourism development [38]. Previous studies have shown that rural public services and rural tourism are feedback loops and codependents, and the two constitute a closely linked and coordinated interaction through the two-way circular flow of materials and energy. Coupling originates from the concept of physics and reflects the strength of interactions and relationships between systems and the state of coordination [39]. The coupling coordination degree model, which captures the transition from uncoordinated to coordinated states between systems, has demonstrated its value and relevance in analyzing the mutual effects and interactions among systems [40]. The coupling coordination degree model has been put into practice to analyze the spatiotemporal coupling coordination between rural tourism and other systems [41–43] and the spatiotemporal coupling coordination between rural public services and other systems [44] and has been widely used in academic research such as in relevant research of urban geography [45], environmental science [46], and tourism [47]. Therefore, it is scientific, objective, and necessary to assess the interactions between rural tourism and rural public services using coupling coordination models.

Currently, the existing literature provides a strong theoretical basis and methodological support for this study. Although relevant studies have utilized the coupling coordination degree model to analyze interrelationships between rural tourism and township construction, rural tourism and agriculture, and rural public services and poverty, there is a lack of sufficient exploration of the coupling coordination relationship between rural public services and rural tourism. Existing studies have also neglected to employ geographic methods to visualize the spatial characteristics and dynamic evolution of the coupling coordination. Additionally, the influencing factors affecting the development of the two degrees of coupled coordination are complex. However, most previous studies examining the coupling coordination relationship between rural public services or rural tourism and other systems have mainly focused on evaluating and comparing coupling coordination degrees and their spatial and temporal distribution characteristics. The suggestions for coupling coordination development based solely on these results may be relatively unconvincing. To address the research gaps in existing research, this paper aims to make contributions and innovations: (1) This paper explores the coupling coordination relationship between the rural public service system and the rural tourism system and analyzes their spatial and temporal differences, starting from their mutual influence. (2) This paper explores the causative factors of the coupling coordination between rural public services and rural tourism and then, objectively proposes strategies for their coordination development.

## 3. Materials and methods

### 3.1 Study area and data sources

Southwestern China, namely Sichuan, Chongqing, Yunnan, Guizhou, and Guangxi (longitude: 97°15′~112°11′E, latitude: 21°04′~34°19′N) [48], which are near the upper borders of the Yangtze River and the Pearl River, exhibit a total area of 1.36 million km2. The study regions exhibit vast rural areas, a complex and diverse topography, offering immense rural tourism resources that include both humanistic and natural elements. More specifically, 56.36% of China’s ethnic minorities originate from and dwell in southwestern China, and their unique ethnic culture has become a crucial tourist resource. In addition, the inclusion of ancient civilization remains, religious culture and other humanistic elements can potentially enhance and enrich rural tourism experiences. Southwestern China plays a significant role in China’s rural tourism development. In 2019, 155 villages, which are in southwestern China which account for approximately 16% of the country, were ranked as China’s most beautiful villages. Additionally, taking that region as an example, the world’s natural and cultural heritage accounted for 25.45% of the country. The region’s total rural tourism revenue exceeded 1,180 billion yuan in 2019, and the rich tourism resources provide a robust foundation for the development of rural tourism in southwestern China. This region is immensely mountainous and hilly, and it exhibits a karst topography, which is unsuitable for economic development. Therefore, it accounts for a large proportion an impoverished areas in China. The southwestern region, being comparatively less developed than the eastern region, demands substantial construction investments to enhance public services such as education, transportation, and ecology. These efforts are necessary to promote the region’s overall development and bridge the development gap between the two regions.

A lower population density also increases the expense of public service construction in rural areas, which hinders the supply of rural public services. Southwestern China exhibits numerous research problems, and because the problems exhibit statistical significance, further research on the topic is necessary. Therefore, using this region as a case study, we can obtain universally applicable conclusions, which can promote the coordinated development of rural public services and rural tourism.

Because tourism was adversely impacted by the coronavirus (Covid-19) pandemic, data from 2020 onwards is excluded. The statistics that we utilized were in this study obtained from the 2012–2019 China Statistical Yearbook, China Rural Statistical Yearbook, China Statistical Yearbook of Culture, Heritage and Tourism, China Statistical Yearbook of Urban and Rural Construction, China Statistical Yearbook of Society, China Statistical Yearbook of Education, official data published by provincial cultural and tourism departments, and provincial national economic and Social Development Statistical Bulletin. We also utilized linear interpolation for the individual years of missing data, we utilized linear interpolation. The data sources can be found in the Data Availability section and S1 File.

### 3.2 Evaluation index system construction

The index system is divided into two systems, namely rural tourism, and rural public services. The establishment of a representative system of indicators facilitates the understanding of the development status and changes among different systems [49]. Drawing on relevant research findings [30, 42–44, 50], the 14th Five-Year Plan for Public Services, and considering the actual conditions in the study area [51], along with scientific and representative data and expert opinions, a comprehensive indicator system for rural public services and rural tourism was formulated. The indicator weights were determined using the entropy value method (Table 1).

**Table 1:** Criteria for classification of coupling coordination levels.

Coupling coordination interval	Level of coordination	Efficacy Comparison	types
[0, 0.5]	On the verge of disorder	U_1_ >U_2_	Rural tourism lagging type
		U_1_ <U_2_	Rural public services lagging type
(0.5, 0.6]	Primary coordination	U_1_ >U_2_	Rural tourism lagging type
		U_1_ <U_2_	Rural public services lagging type
(0.6, 0.8]	Intermediate coordination	U_1_ >U_2_	Rural tourism lagging type
		U_1_ <U_2_	Rural public services lagging type
(0.8, 1]	Senior coordination	U_1_ >U_2_	Rural tourism lagging type
		U_1_ <U_2_	Rural public services lagging type

Existing scholars have predominantly created public service subsystems by categorizing them along dimensions such as infrastructure, education, health, culture, and social security [38, 44]. Given that environmental quality is another important factor in tourism and financial development [52], we incorporate environmental protection into the rural public service subsystem. More specifically, the rural public service indicator system includes 17 specific indicators in six sub-system layers: infrastructure, elementary education, health care, environmental protection, entertainment culture, and social security. Infrastructure is an indispensable material foundation in the process of rural development. This paper measures infrastructure set-up from the perspective of residents’ daily lives, which mainly includes three specific indicators: the number of rural sanitary toilets [38], road area per capita [50], and rural water supply penetration rate [53]. Elementary education mainly reflects the human and financial resources invested in the development of basic education from the perspective of teachers and students, and that observation includes three specific indicators: the teacher-student ratio in primary schools [54], the teacher-student ratio in junior middle schools [54], and the illiteracy rate of the population over 15 years of age [55]. Health care mainly includes medical conditions in terms of various institutions, facilities, and workforce, and the number of health employees per 10,000 people [55] and the number of health clinics per 10,000 people [53] were selected for characterization. Environmental protection is reflected in terms of investment in ecological and environmental protection services, facilities, and the effectiveness of environmental protection and is characterized by the rural household waste disposal input [4], the village green space rate [4], and the village sewerage inputs [4]. Entertainment culture mainly reflects the government’s investment in recreational and cultural facilities and the ability of the government and society in organizing local cultural activities. Three specific indicators were selected to characterize the number of rural cultural stations [53], the number of villages’ cultural events [4], and the number of exhibitions held in villages [4]. Social security plays an important role in ensuring that the livelihoods of residents are improved, maintaining social equity, and promoting people’s well-being. The average standard of subsistence minimum for rural residents [54], the rural residents covered by social pension insurance [53], and the number of rural support institutions [54] were selected to characterize social security.

When it comes to rural tourism, scholars generally create indicator systems that encompass the subsystems of development level, industrial scale, resource base, and support conditions. In this paper, the rural tourism index system includes 10 specific indicators in three subsystem layers: level of development, resource base, and support conditions. The level of development reflects the overall development of rural tourism, and the annual income of rural tourism [42], rural tourism visits [30], and the per capita net income of rural residents [56] are selected to characterize the development level. The resource base is an important tourism attraction for rural tourism, and as many rural tourism sites in China rely on nationally published model sites and scenic area ratings to carry out their development, the number of A-class scenic spots [30], number of National-level Leisure Agriculture and Rural Tourism Demonstration Counties (Sites) [57], number of China’s Beautiful Leisure Rural [57], and number of National “One Village, One Product” Model Village [57] can be better characterized. Support conditions reflect the service capacity of rural tourism, and the number of travel agencies [41], the number of tourism workers [57], and road passenger turnover [58] were chosen to characterize them.

### 3.3 Methods

#### 3.3.1 Data standardization

Since the selected indicators have different scales and each indicator has a positive or negative impact on the system, they cannot be easily used or compared. Therefore, the raw data were standardized using the ‘extreme difference’ standardization method.

$$\text{Positive}\;\text{indicators}:Z_{\text{ij}}=\frac{x_{\text{ij}}-\text{min}(x_{ij})}{\text{max}(x_{ij})-\text{min}(x_{ij})}+0.0001$$ (1)

$$\text{Negative}\;\text{indicators}:Z_{\text{ij}}=\frac{\text{max}(x_{ij})-x_{ij}}{\text{max}(x_{ij})-\text{min}(xij)}+0.0001$$ (2)

where *x*_ij_ and *Z*_ij_ are the original and standardized values of the jth indicator in year I, respectively. To avoid invalid data in the operation process, the data is panned by 0.0001 in the standardization process.

#### 3.3.2 Comprehensive evaluation model

The comprehensive evaluation index of rural public services and rural tourism is calculated by using the weighted sum of weights and indicators. Before calculating the comprehensive evaluation index of the two systems, it is necessary to assign weights to each index. This paper uses the entropy method to objectively assign weights to indicators, which can effectively avoid the subjective arbitrariness caused by the subjective weighting method and has been widely used in multidisciplinary fields [13, 59, 60], and its calculation results are scientific and reliable. The specific calculation steps are as follows:

Step 1. The weighting of each indicator was transformed:
$$p_{ij}=Z_{ij}/\sum_{i=1}^{n}Z_{ij}$$ (3)
Step 2. Calculate the entropy value of for the jth indicator *e*_*j*_:
$$e_{j}=-\frac{1}{\text{ln}\;n}\sum_{i=1}^{n}p_{ij}\;\text{ln}(p_{ij})$$ (4)
Step 3. Calculate the degree of variation of the jth indicator g_*j*_:
$${\text{g}}_{j}=1-e_{j}$$ (5)
Step 4. The weight of the jth indicator was calculated w_j_:
$$w_{j}=g_{j}/\sum_{j=1}^{m}g_{j}$$ (6)
Step 5. Calculate the comprehensive evaluation index:
$$U_{1,2}=\sum_{\text{j}=1}^{\text{m}}w_{\text{j}}\times Z_{\text{ij}}$$ (7)
  where *U*_1,2_ denotes the comprehensive evaluation index, w_j_ denotes the weight of the indicator, and *Z*_*ij*_ denotes the value obtained after standardization of indicator j in year i of a province and after panning.

#### 3.3.3 Coupling coordination degree model

The coupling coordination degree model can quantitatively evaluate the degree of coordination of coupling between two or more systems and has become an effective evaluation method and research tool for studying the degree of overall balanced development of a region. Based on the coupling evaluation model in physics, this paper introduces the coupling coordination degree model (CCDM) [61] to measure the coordination relationship between rural public services and rural tourism, considering the characteristics of the object. The calculation steps are as follows:

Step 1. includes calculating the coupling degree. Coupling degree refers to the phenomenon of two or more systems interacting with each other through an interaction mechanism. The closer the two systems are, the higher the coupling degree between them, with the following equation:
$$C=\sqrt{\frac{U_{1}U_{2}}{{\left(\frac{U_{1}+U_{2}}{2}\right)}^{2}}}=\frac{2\sqrt{U_{1}U_{2}}}{U_{1}+U_{2}}$$ (8)
  where *C* denotes the coupling degree and assumes the value of [0, 1]. When the resultant value approximates 1, the coupling degree is enhanced. *U*_1_ and *U*_2_ denote the comprehensive evaluation indices affecting rural public services and tourism, respectively.
Step 2. includes calculating the coupling coordination degree. The coupling degree focuses on the ability of the systems to evolve in tandem. High coupling can also occur when all systems are at a low level of development, but its numerical magnitude makes it difficult to describe the interaction effects between the systems, and the true state of coordination cannot be revealed. As a complement to the coupling degree, the coupling coordination degree can distinguish between a low level of high coupling and a high level of coupling in terms of both correlation and coordination and can also truly reflect the degree of virtuous mutual feedback between systems with coupling relationships, and it utilizes the following formula:
$$D=\sqrt{C\times T}$$ (9)
$$T=\alpha U_{1}+\beta U_{2}$$ (10)
  where *D* denotes the coupling coordination degree, and *T* denotes the comprehensive coordination index. Additionally, α and β denote the unevaluated coefficients, which represent the contribution of rural public services and rural tourism, respectively. Since rural public services and rural tourism are both crucial systems, their importance and contribution are considered to be equal. Thus, both the α and β levels were set at 0.5. To effectively characterize the coordinated development of rural public services and rural tourism, it is essential to consider the characteristics, features, and laws that regulate both systems. Particularly, referencing the study conducted by Ma, M. Y. et al [52], the coupling coordination degree is categorized into four distinct groups (Table 2).

**Table 2:** Evaluation index system and weights of rural public services and rural tourism.

System layer	Subsystem layer	Specific indicators	Weights/Attributes
Rural public services	Infrastructure	Number of rural sanitary toilets	0.0817/+
		Road area per capita (m^2^)	0.0767/+
		Rural water supply penetration rate (%)	0.0250/+
	Elementary education	Teacher-student ratio in primary schools (%)	0.0291/+
		Teacher-student ratio in junior middle schools (%)	0.0371/+
		Illiteracy rate of population over 15 years of age (%)	0.0252/-
	Health care	Number of health personnel per 10,000 population	0.0375/+
		Number of health clinics per 10,000 people	0.0430/+
	Environmental protection	Rural household waste disposal input (10,000 CNY)	0.0688/+
		Village green space rate (%)	0.0388/+
		Village sewerage inputs (10,000 CNY)	0.0963/+
	Entertainment culture	Number of rural cultural stations	0.1352/+
		Number of village cultural events	0.0628/+
		Number of exhibitions held in villages	0.0486/+
	Social security	Average standard of subsistence minimum for rural residents (CNY)	0.0428/+
		Rural residents covered by social pension insurance (10,000 people)	0.0611/+
		Number of rural support institutions	0.0902/+
Rural tourism	Level of development	Annual income of rural tourism (100,000,000 CNY)	0.1367/+
		Rural tourism visits (100,000,000 people)	0.1182/+
		Per capita net income of rural residents (CNY)	0.0560/+
	Resource base	Number of A-class scenic spots	0.1064/+
		Number of National-level Leisure Agriculture and Rural Tourism Demonstration Counties(Sites)	0.0549/+
		Number of China’s Beautiful Leisure Rural	0.1377/+
		Number of National “One Village, One Product” Model Village	0.0860/+
	Support condition	Number of travel agencies	0.0867/+
		Number of tourism workers	0.1113/+
		Road passenger turnover (100,000,000 person-km)	0.1062/+

**Note**: The weights are calculated by the entropy method to determine the importance of the evaluation indicators; the direction of the attributes is based on the positive and negative impact effects.

#### 3.3.4 GeoDetector

Causative factors play a significant role in influencing the coupling coordination development between rural public services and rural tourism. The GeoDetector, comprising a set of statistical methods, proves valuable in detecting spatial differentiation and revealing the causative factors that impact this relationship [62]. Unlike conventional model-building approaches, this method is less constrained by assumptions. It excels in detecting interactions between variables, not restricted to predetermined multiplicative interactions typically associated with econometrics. Due to its accurate measurement of causative factors, GeoDetector finds wide application in various fields. Thus, to reveal the causative factors influencing the coupling coordination between rural public services and rural tourism, this study employed a GeoDetector analysis, utilizing the freely available GeoDetector software (http://www.GeoDetector.org/). The following formula was utilized:

$$q=1-\frac{1}{N\delta^{2}}\sum_{i=1}^{L}Ni\delta \begin{array}{c} 2\\ i \end{array}$$ (11)

where *N* denotes the full sample size of the study area. Additionally, $\delta_{\text{i}}^{2}$ denotes the variance of the indicator, and *i* denotes the partition, while *L* denotes the number of partitions. What is more, *q* values assume values in the [0, 1] range, and their magnitudes reflect the degree of spatial differentiation. When the *q* value is large, the influence that the factor exerts on the coupling coordination becomes great.

## 4. Results

### 4.1 Spatial and temporal characteristics of the integrated development

#### 4.1.1 Characteristics of the time series curves

**Fig 1:**
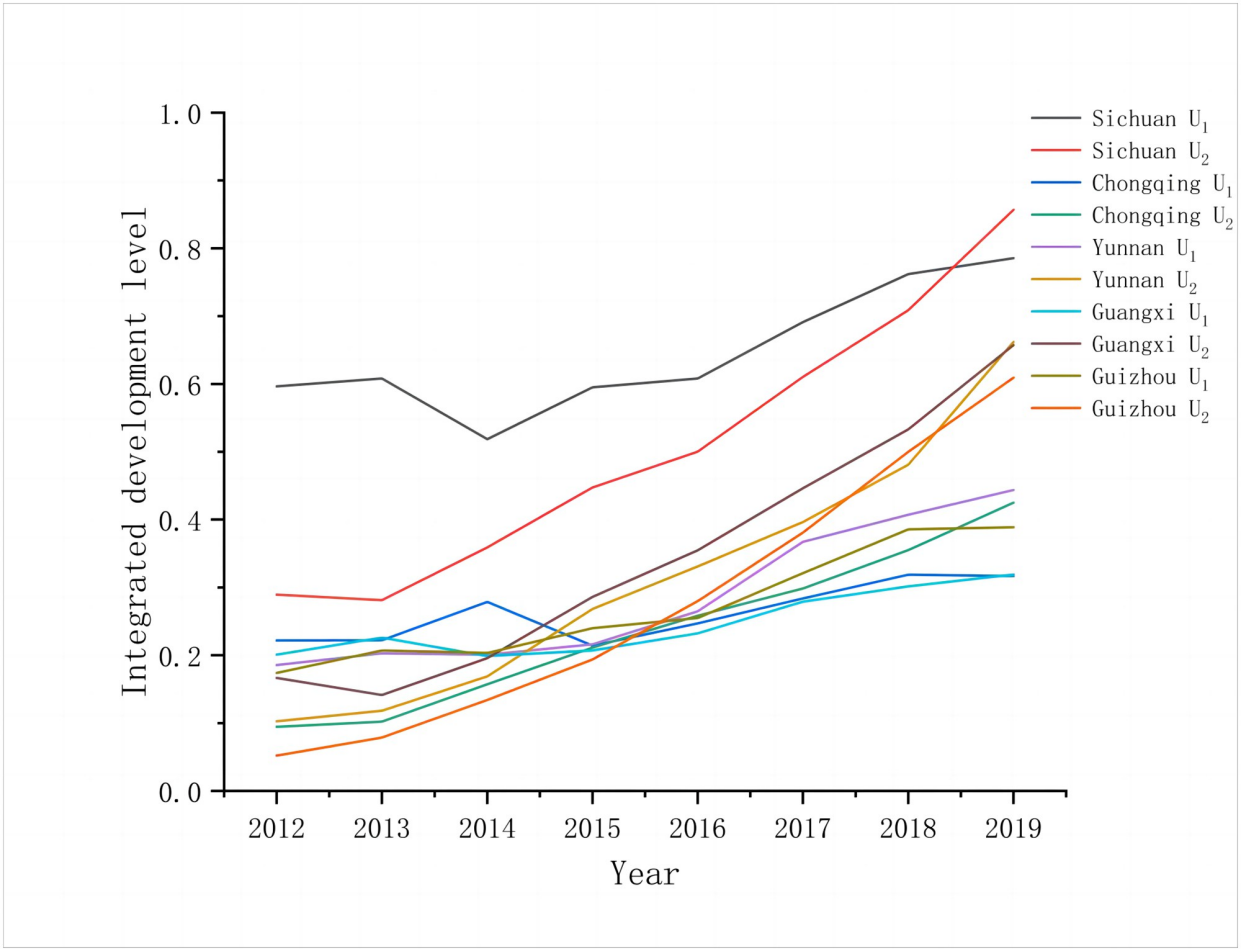
Integrate development level of rural public services and rural tourism in southwestern China, 2012–2019.

To calculate the results of the integrated development level, this study utilized the panel data that was collected from 2012 to 2019, and it utilized a comprehensive evaluation model (Fig 1). In this regard, Fig 1 indicates that rural public services exhibit an upward trend, from a slight fluctuation to a rapid increase. In southwestern China, the integrated development index of rural public services fluctuated from 2012 to 2016, while in 2017 it began to increase quickly. The introduction of the 13th Five-Year Plan for Promoting Equalization of Basic Public Services, which was developed in 2017, has signalled the construction of a novel development pattern in the rural public services context. Subsequently, the policy radiated to rural areas more extensively. Thus, from 2017 onwards, the integrated development index of rural public services became significantly higher.

In southwestern China, the integrated development index of rural tourism indicates immense growth compared with that of rural public services, with the average annual growth rate attaining 27.01%. From 2012 to 2019, the rural tourism development index of Sichuan, Chongqing, Yunnan, and Guangxi increased by 1.96, 3.5, 5.44, and 3.94 times, respectively. Notably, the rural tourism development and resource base pertaining to Guizhou’s rural tourism index indicates a gradual enhancement, and its supporting conditions are increasingly robust. This indicates a positive development trend. Since the release of the National Rural Tourism Development Outline (2009–2015) and the implementation of targeted poverty alleviation and rural revitalization, tourism has become a crucial method of stimulating urban-rural consumption, enhancing farmers’ income, and revitalizing rural industries. It is also worth noting that the role of policy guidance has been continuously strengthened due to enhanced industrial policies.

The time series curves of the integrated development level of the two systems (study period: 2012–2019) indicate an upward trend. However, the integrated development index of rural public services exceeded that of rural tourism in 2012, and in 2019, it lagged. Thus, the development of rural public services is slower than that of rural tourism. This observation reflects the long-term urban-rural dual structure. Furthermore, it indicates other rural development problems and those rural public services exert a highly significant promotion effect on rural tourism. The influence of rural tourism and the promotion effect that it exerts on rural public services are yet to be revealed. Therefore, the quality and development of rural public services require further enhancement.

#### 4.1.2 Characteristics of the spatial pattern

To observe regional differences, the study divided the southwestern China region into five development levels based on the integrated development level of the two systems and existing research results [30]. The following are the observed development level categories:

- U_1_, U_2_ ∈ [0, 0.15]: Low development level
- U_1_, U_2_ ∈ (0.15, 0.30]: Lower development level
- U_1_, U_2_ ∈ (0.30, 0.45]: Medium development level
- U1, U2 ∈ (0.45, 0.60]: High development level
- U_1_, U_2_ ∈ (0.6, 1]: High development level

**Fig 2:**
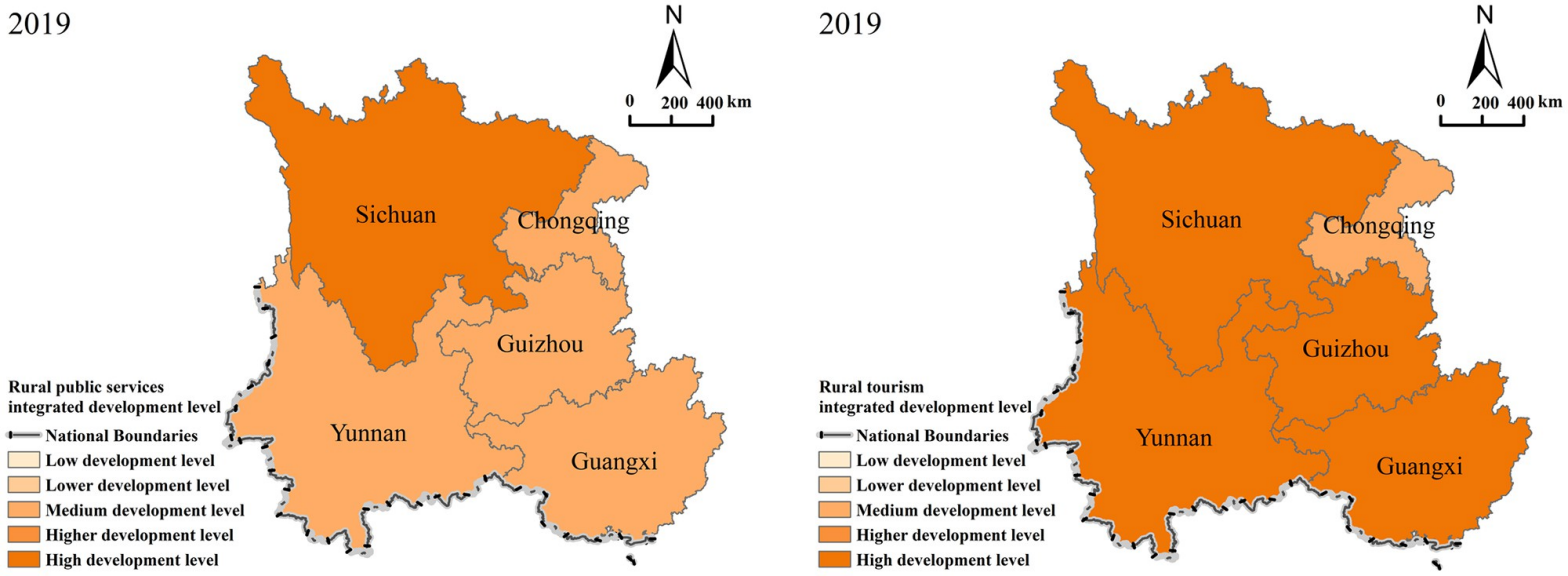
Spatial pattern of integrated development levels of rural public services and rural tourism in southwestern China, 2019. This map is made according to the standard map provided by the Natural Earth (http://www.naturalearthdata.com/), and the figure is similar to the original image, but not the same.

To examine the recent progress of the two systems in southwestern China during the study period, the researchers selected 2019 as the focal year to analyze the spatial patterns representing the integrated development of these two systems (Fig 2).

1. The development level of rural public services. The results indicate that in southwestern China, the overall development level of rural public services is generally weak, with Chongqing, Yunnan, Guangxi, and Guizhou exhibiting medium development levels of 0.317, 0.444, 0.319, and 0.389, respectively. The preceding observation may be explained by Sichuan’s substantial economic endowment, and by the ideal rural public services development conditions that characterize the region.
2. The development level of rural tourism. By contrast, rural tourism exhibits a high development level (Fig 2). Sichuan exhibits the highest development level (0.857), and Chongqing exhibits the lowest development level (0.425). Chongqing’s mid-level development indicates that all other provinces attained a high level of development. Rural tourism in Yunnan, Guangxi, and Guizhou is highly valued by the government because of its rich resources and unique cultural charm and has been vigorously developed, especially after the introduction and implementation of the strategy of whole-area tourism and rural revitalization. Based on the spatial pattern characteristics of the integrated development level, it can be observed that the two systems exhibit uneven regional development. Therefore, to achieve high-quality rural development, governments must comprehensively enhance both rural public services and rural tourism. This involves a continuous strengthening of policy guidance, regulation, and financial investment. By adopting such measures, the potential for substantial progress in the integrated development of these two systems can be realized.

### 4.2 Spatial and temporal differentiation in the coupling coordination

#### 4.2.1 Temporal characteristics of coupling coordination

**Fig 3:**
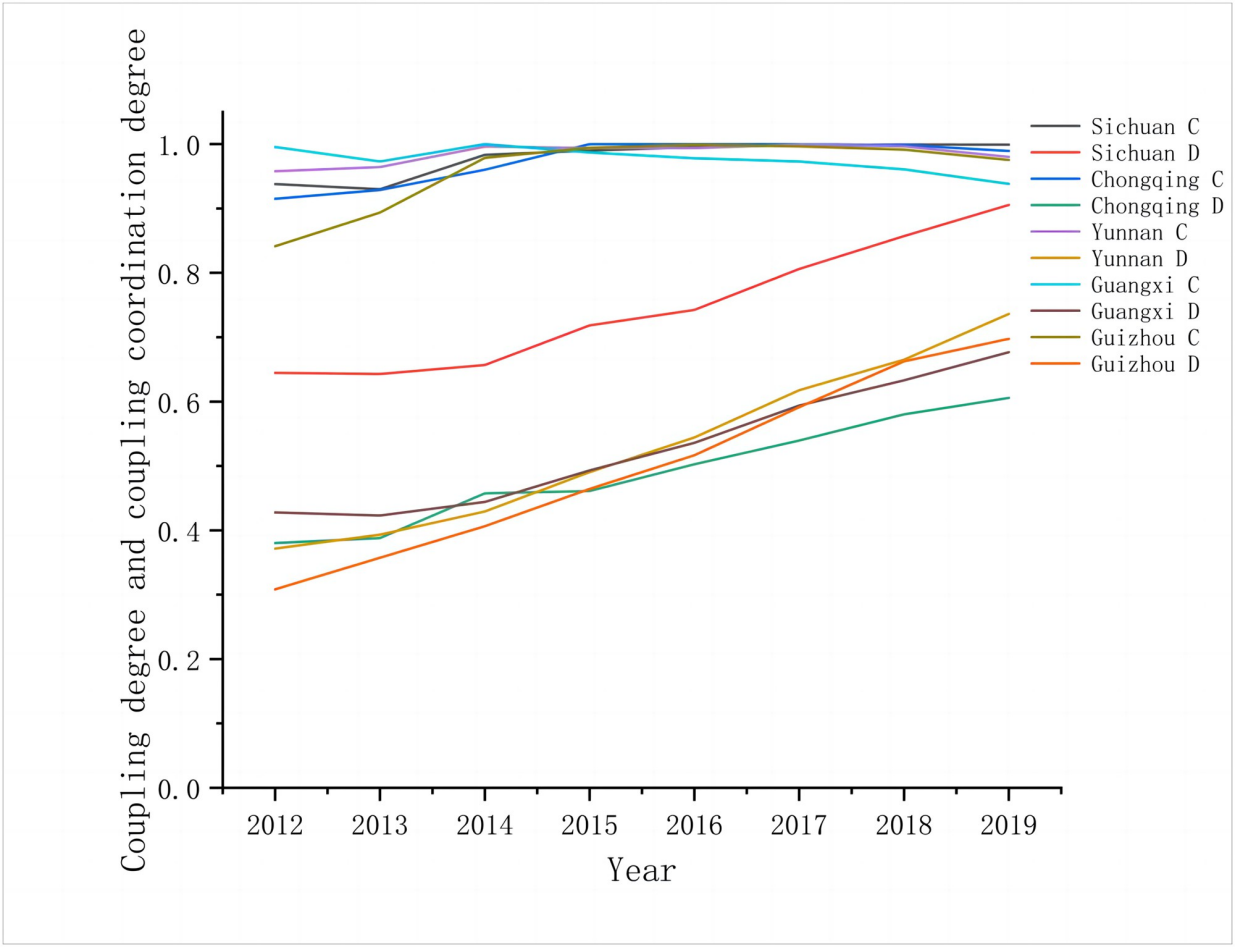
Comparison of coupling degree and coupling coordination degree between rural public services and rural tourism in southwestern China, 2012–2019.

This time-series-based study analyzed the coupling coordination degree of the two systems, and it employed the coupling coordination degree model, which can effectively detect the change between rural public services and rural tourism in time series (Fig 3).

1. From 2012 to 2016, the coupling coordination of the two systems exhibited a gradual but slow increase. Chongqing, Yunnan, Guangxi, and Guizhou transited from near dissonance to the primary coordination stage, and Sichuan maintained the intermediate coordination stage. The preceding observation indicates that at this stage, the two systems still exhibited low-level coupling coordination and had not attained the optimal coupling state. China’s struggle against poverty is currently in its infancy, and as a result, the rural infrastructural, educational, medical, and environmental protection facilities in southwestern China are underdeveloped. Rural tourism is also still in its infancy and has little development.
2. From 2017 to 2019, the coupling coordination of the two systems came to a sharp increase. Chongqing, Yunnan, Guangxi, and Guizhou transitioned from the primary stage to the intermediate one, and Sichuan transitioned from the intermediate level into the advanced coordination stage. The mutual feedback between the two systems is successively enhanced. Along with the introduction of the 2017 “13th Five-Year Plan for Promoting the Equalization of Basic Public Services”, “Action Plan for Promoting the Quality and Upgrading of Rural Tourism Development (2017)”, “Notice on the Issuance of the Guidance on Promoting the Sustainable Development of Rural Tourism” (No. [2018] 98), and other associated policies, the development of the policy system for rural public services and rural tourism has exhibited considerable results, and the development of rural public services and rural tourism has progressed considerably. Therefore, the coupling and coordination of the two systems has increased rapidly.

The detailed comparison of the integrated development indices of the two systems is indicated in Table 3, and the coupling coordination development can be divided into two types, namely rural tourism lagging type and rural public service lagging type. According to Table 3, it is apparent that all southwestern China exhibited tourism lagging types during the first few years pertaining to the study period. This observation indicates that during the early stage, rural public services development exceeds rural tourist development, which provides a robust foundation for the high-quality development of rural tourism. At a later stage, the total southwestern China area became the public services lagging type, which indicates that between 2012 and 2019, the rural tourism and rural public services that characterize southwestern China have accelerated. Thus, from 2012 to 2019, rural tourism had a sharp increase and the level of rural public services turned lower than that of rural tourist development. This is because public service facilities were developed earlier in the early phase, and rural tourism is still in the participation stage of the life cycle of the tourist destination. Rural tourist development may have sufficient public services, such as material support, rural roads, environmental health, and other amenities, to meet the needs of rural tourist facilities. However, as the number of rural tourism visitors continues to increase due to rising tourist demand, public services face challenges in coping with the supply pressure brought about by the rapid development of rural tourism.

**Table 3:** Results of measuring the coupling coordination relationship between rural public services and rural tourism in southwestern China.

District	Year	U_1_	U_2_	C	D	Coherence level—type
Sichuan	2012–2016	0.597~0.608	0.289~0.500	0.938~0.995	0.645~0.742	Intermediate coordination—rural tourism lagging type
	2017–2018	0.691~0.762	0.610~0.709	0.998~0.999	0.806~0.857	Senior coordination—rural tourism lagging type
	2019	0.786	0.857	0.999	0.906	Senior coordination—rural public services lagging type
Chongqing	2012–2015	0.222~0.214	0.094~0.211	0.915~0.999	0.380~0.461	On the verge of disorder—rural tourism lagging type
	2016–2018	0.247~0.319	0.258~0.355	0.999~0.998	0.503~0.580	Primary coordination—rural public services lagging type
	2019	0.316	0.425	0.989	0.606	Intermediate coordination—rural public services lagging type
Yunnan	2012–2014	0.186~0.201	0.103~0.169	0.958~0.996	0.372~0.429	On the verge of disorder—rural tourism lagging type
	2015	0.216	0.268	0.994	0.491	On the verge of disorder—rural public services lagging type
	2016	0.265	0.331	0.994	0.544	Primary coordination—rural public services lagging type
	2017–2019	0.367~0.444	0.396~0.662	0.999~0.980	0.618~0.736	Intermediate coordination—rural public services lagging type
Guangxi	2012–2014	0.201~0.199	0.167~0.196	0.996~0.999	0.428~0.444	On the verge of disorder—rural tourism lagging type
	2015	0.207	0.286	0.987	0.493	On the verge of disorder—rural public services lagging type
	2016–2017	0.232~0.279	0.355~0.446	0.978~0.973	0.536~0.594	Primary coordination -rural public services lagging type
	2018–2019	0.302~0.319	0.533~0.657	0.961~0.938	0.633~0.677	Intermediate coordination—rural public services lagging type
Guizhou	2012–2015	0.174~0.240	0.052~0.194	0.841~0.994	0.308~0.464	On the verge of disorder—rural tourism lagging type
	2016–2017	0.255~0.321	0.280~0.381	0.999~0.996	0.517~0.591	Primary coordination—rural public services lagging type
	2018–2019	0.386~0.389	0.500~0.609	0.992~0.975	0.663~0.698	Intermediate coordination—rural public services lagging type

#### 4.2.2 Spatial distribution of coupling coordination

**Fig 4:**
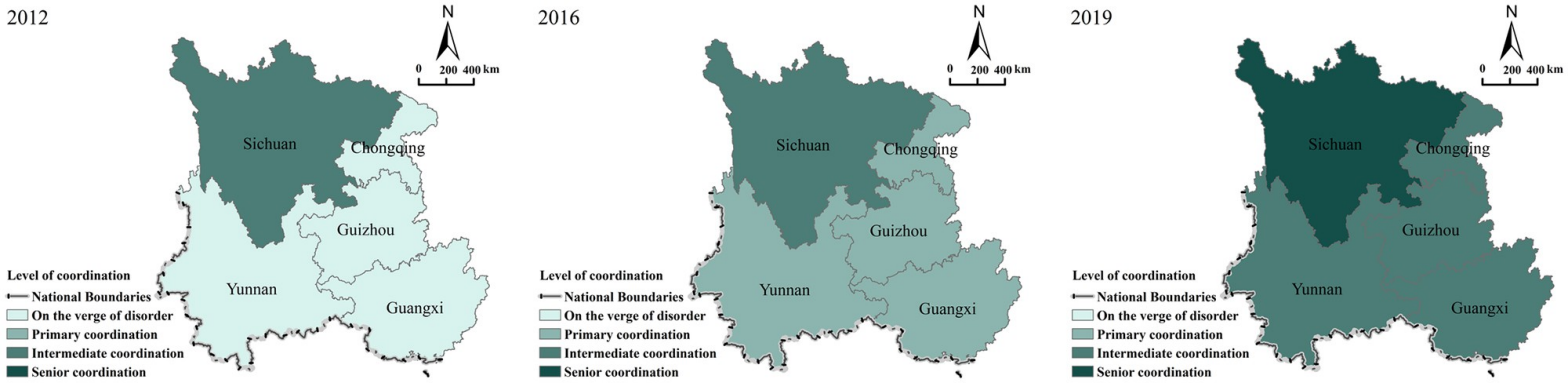
Spatially divergent patterns of coupling coordination between rural public services and rural tourism in southwestern China. This map is made according to the standard map provided by the Natural earth (http://www.naturalearthdata.com/), and the figure is similar to the original image, but not the same.

This study, which focuses on three annual periods, namely 2012, 2016, and 2019, spatially visualized the types of coordination (Fig 4). Thus, it revealed the spatial combination of different coupling coordination degrees, and it considered the temporal difference. Table 3 and Fig 4 indicate that in southwestern China, the values of the coupling coordination between rural public services and rural tourism (study period: 2012 to 2019) range from 0.308 to 0.906. In this regard, an apparent spatial heterogeneity is observed, with noticeable differences between Sichuan and other provinces. However, the development of each province becomes more synchronized. In 2012, Sichuan was the first province to transit into the intermediate coordination stage, whereas other provinces were mainly in the near-dissonance stage. Chongqing, Yunnan, Guangxi, and Guizhou successively transited into the primary coordination stage in 2016, whereas Sichuan remained in the intermediate coordination stage. Chongqing, Yunnan, Guangxi, and Guizhou attained the intermediate coordination stage in 2019, whereas Sichuan attained the advanced stage.

If the calculation of the spatial coupling coordination level of southwestern China is extended, we can observe the following:

1. Sichuan’s coupling coordination level considerably exceeded that of the other provinces for eight consecutive years. Sichuan has achieved certain results in rural public services, relying on its strong economic foundation and national policy support, while the government places importance on cultural branding and tourism labelling, using the potent allure of brand IP to draw tourists and promote the high-quality development of rural tourism.
2. Chongqing’s coupling coordination level exceeded that of Yunnan and Guizhou in 2012, whereas after this period, it gradually became lower than that of other provinces, and that indicates a lagged development. The explanation for this is that Chongqing is close to Sichuan, and because of Sichuan’s unique characteristics and market competitiveness, Sichuan has developed an "image masking" of Chongqing, which unavoidably weakens Chongqing’s image for rural tourism.
3. Guizhou’s coupling coordination level increased sharply, and in 2019, its value increased by 127%. With the advantages of its rich and unique tourism resources, Guizhou began to develop tourism vigorously, realizing the rapid improvement of branding, scale, and standardization of rural tourism, as well as the rapid improvement of infrastructure construction and residents’ living standards. The relationship between rural public services and rural tourism has also become more favorable.

### 4.3 Analysis of the causative factors associated with the coupling coordination

The coupling coordination development between rural public services and rural tourism is influenced by various factors. In this study, based on previous research results [63, 64], the geographic probe method was chosen to conduct an actual situation analysis. The study focused on detecting the influence of four driving factors: economic causative force, industrial causative force, resource attractiveness, and service guarantee. More specifically, the level of economic development is the material guarantee for the systematic coupling and coordinated development of the two systems, providing capital support for the optimal allocation of public resources and the further development of the service sector [65]. To measure the economic causative forces, the annual income of rural tourism and the per capita net income of rural residents were utilized. In economically backward and less developed regions, tourism often serves as a common niche industry [66] playing a crucial role in promoting coordinated regional development. This study utilized the number of rural tourism visits and the number of travel agencies as indicators. Resource attractiveness, on the other hand, serves as the underlying cause of tourist motivation, influencing tourist behavior [66], and also plays a decisive role in inter-industry coordination [63]. The number of National-level Leisure Agriculture and Rural Tourism Demonstration Counties (Sites) and the number of China’s Beautiful Leisure Rural destinations were utilized to measure the level of resource attractiveness. With the growing demand for public services, the availability of public facilities and related services is crucial [66]. The number of rural sanitary toilets (Infrastructure), the teacher-student ratio in junior middle school (Elementary education), the number of health clinics per 10,000 people (Health care), the number of rural household waste disposal inputs (Environmental protection), the number of rural cultural events (Entertainment culture), and the average standard of the subsistence minimum for rural residents (Social security) were utilized. Based on the above, the level of service guarantee was measured. Furthermore, to discretize the continuous variables associated with the indicators and to achieve hierarchical processing, we utilized SPSS 25.0. Finally, we calculated the single-factor influence degree and factor-interaction degree that each driving factor exerted on the coupling coordination development of rural public services and on that of rural tourism, and we obtained relevant data for southwestern China (Tables 4 and 5).

**Table 4:** Results of the causative factor of coupling coordination development of rural public services and rural tourism.

Causative factor	Detection indicators	q
Economic causative force	Annual income of rural tourism (*X*_1_)	0.785
	Per capita net income of rural residents (*X*_2_)	0.570
Industrial causative force	Number of rural tourism visits (*X*_3_)	0.801
	Number of travel agencies (*X*_4_)	0.385
Resource attractiveness	Number of National-level Leisure Agriculture and Rural Tourism Demonstration Counties(Sites) (*X*_5_)	0.715
	Number of China’s Beautiful Leisure Rural (*X*_6_)	0.552
Service guarantee	Number of rural sanitary toilets (*X*_7_)	0.516
	Teacher-student ratio in junior middle school (*X*_8_)	0.568
	Number of health clinics per 10,000 people (*X*_9_)	0.504
	Rural household waste disposal inputs (*X*_10_)	0.807
	Number of village cultural events (*X*_11_)	0.705
	Average standard of subsistence minimum for rural residents (*X*_12_)	0.497

**Table 5:** Multi-factor interaction detection results of coupling coordination development between rural public services and rural tourism.

	*X*_1_	*X*_2_	*X*_3_	*X*_4_	*X*_5_	*X*_6_	*X*_7_	*X*_8_	*X*_9_	*X*_10_	*X*_11_	*X*_12_
*X*_1_	0.785											
*X*_2_	0.897	0.570										
*X*_3_	0.871	0.899	0.801									
*X*_4_	0.907	0.719	0.941	0.385								
*X*_5_	0.948	0.773	0.939	0.782	0.715							
*X*_6_	0.892	0.664	0.881	0.710	0.842	0.552						
*X*_7_	0.911	0.901	0.945	0.772	0.857	0.888	0.516					
*X*_8_	0.945	0.794	0.889	0.789	0.845	0.907	0.806	0.568				
*X*_9_	0.914	0.904	0.940	0.835	0.884	0.966	0.650	0.792	0.504			
*X*_10_	0.907	0.919	0.933	0.895	0.924	0.943	0.896	0.895	0.908	0.807		
*X*_11_	0.927	0.869	0.915	0.781	0.882	0.960	0.862	0.831	0.773	0.888	0.705	
*X*_12_	0.873	0.713	0.861	0.650	0.826	0.626	0.858	0.922	0.941	0.934	0.935	0.497

Using one-factor probing, a quantitative analysis of the driver-by-driver effect was conducted and based on that, we visually observed the influence that the q magnitude exerted on the coupled coordination of rural public services and rural tourism, and we considered the case of each driver.

1. Economic causative force. Table 4 indicates that the q-value of the annual income of rural tourism is 0.785, and the q-value of the per capita net income of rural residents is 0.570, which indicates that it significantly enhances the coupling coordination of the two factors. The impact that is occasioned by the economic driving force is mainly reflected in the increased income and industry levels. In rural areas, the infusion of high-value capital can significantly contribute to fostering the growth of the local rural tourism industry. This investment aims to promote high-quality development and enhance public services while simultaneously bolstering the advancement of the two key factors. Economic factors serve as crucial indicators and causative forces for the coupled and coordinated development of the two systems. They are necessary prerequisites for rural tourism development and the enhancement of rural public service supply.
2. Industrial causative force. According to the results of the current study, the industrial causative force exerts considerable causative effects on the coordinated development of the two systems. More specifically, the causative effect that is associated with rural tourism visits exhibits a larger influence (q value: 0.801). The number of travel agencies exhibits a *0*.*385* q value, which is smaller than that of other detection indicators. However, its value exhibits a significant influence. Due to industrial structure adjustment, rural tourism transited from operating extensively to gradually exhibiting highly concentrated operation. The combination of multi-industry factors and multi-sectoral ones can enhance the rural public service, and it can promote the coordinated development of both systems.
3. Resource attractiveness. The q-values associated with the number of National-level Leisure Agriculture and Rural Tourism Demonstration Counties (Sites) and the number of China’s Beautiful Leisure Rural are 0.715 and 0.552, respectively. The results indicate that the role of resource attractiveness is crucial for coordinated development. National-level Leisure Agriculture and Rural Tourism Demonstration Counties (Sites) and China’s Beautiful Leisure Rural crucially facilitate rural tourism development. By promoting the enhancement and optimization of local rural public services and stimulating the local economy, there is an influence on the coordinated development that occurs as part of the coupling of the two systems. Additionally, this can lead to an increase in tax revenue for the region.
4. Service guarantee. The q-values of the six indicators that reflect the service guarantee range from 0.497 to 0.807, which indicates that the service guarantee exerts a strong causative effect on the coordinated development of the two systems. Rural public services enhance the welfare and development of rural residents and promote rural tourism. Regarding the six indicators, the teacher-student ratio in junior middle schools emerges as a significant driving force, suggesting that improving elementary education can effectively foster the development of skilful individuals in rural tourism. Furthermore, the presence and quality of service facilities, such as rural sanitary toilets, waste disposal facilities, and health clinics, have a significant impact on rural tourism development. These indicators can enhance tourists’ destination evaluation and influence their choice of a tourist destination.

The level of coupling coordination development is influenced by a combination of factors. Multi-factor interaction detection was utilized to comprehensively explore the influence of the causative factors. Table 5 shows that the multi-factor interaction drivers have more significant effects compared to the single factor. More specifically, the study identified the number of health clinics per 10,000 people surpassing the number of China’s Beautiful Leisure Rural as the strongest interaction driver, with a q-value of 0.966. This observation highlights the considerable impact of healthcare infrastructure and beautiful countryside construction on the coordination between rural public services and rural tourism.

The second strongest impact is seen in the interaction between the number of village cultural events and the number of China’s Beautiful Leisure Rural, along with the annual income of rural tourism and the number of National-level Leisure Agriculture and Rural Tourism Demonstration Counties (Sites), with q-values of 0.960 and 0.948, respectively. Effective rural recreational and cultural services not only enrich the leisure time of rural residents and enhance their well-being but they are also influenced by the various recreational and cultural service demands from potential tourists. This resource-sharing model provides tourists with abundant rural cultural and tourism activities, which can provide an endogenous impetus to the rural economy and promote the coordinated development of both factors. The industrial development level and resource base are equally significant in the context of coupling coordination between the two factors, indicating that the interaction between the economy and resource elements can enhance the quality of the rural tourism industry supply and that of rural public services.

## 5. Discussion

Due to social and natural conditions, rural areas in developing countries often face challenges in terms of economic development and the welfare of their inhabitants. Internal development in these regions is often uncoordinated, particularly evident in the southwest region of China. To address this issue, this paper focuses on Southwest China as a case study and integrates the concept of new development with the understanding of system theory to better reveal the coupling coordination relationship between rural public services and rural tourism and its causative factors. The general characteristics, patterns, and mechanisms identified in the study can serve as good references for the development of rural areas in other developing countries around the world.

In line with existing research [30, 67], the overall development of rural public services and rural tourism in southwest China has been on the rise. However, this study highlights a widening gap between the two is widening, with rural tourism developing at a faster pace than rural public services and rural tourism has undergone a transition during the study period. It has changed from the rural tourism lagging type to the rural public services lagging type during the study period, which affects the coupling coordination between the two. The possible reason for this change can be attributed to the Chinese government’s increased investment in rural development during the Central Rural Work Conference in 2017. This boost in investments has clarified the goal and task of implementing the rural revitalization strategy, basic education, habitat improvement, and medical services, which has enabled the level of rural public services to significantly improve. However, the long-standing constraints on rural economic development and the "urban over rural" phenomenon [54] still exist, as well as the limited construction of villages and local government’s financial resources. There are still some general problems in the provision of rural public services. Therefore, in the future, continuing to promote the continuous improvement of rural public services will remain a top priority for the coupling coordination development of both.

The causative factors selected in the text drive the coupling coordination of the two systems to a greater extent, but also with variability. Among the single-factor probes, the rural household waste disposal input was the most significant driver. According to Huang, Z et al. [38], the results of this study are corroborated by the fact that improving the quality of the ecological environment also improves the level of public services and promotes tourist development. The strongest interaction driver identified in the multi-factor interaction was the combination of the number of health clinics per 10,000 people and the number of China’s Beautiful Leisure Rural, which aligns with the findings of previous studies [68]. Substantial policy and financial investments in public health services and tourism are beneficial for expanding the scale of demand for rural tourism and rural public services. Additionally, these investments can foster a clustering effect for industrial factors such as rural tourism and rural public services, thereby promoting their overall development and coordination.

Based on the above analysis, this study offers the following proposals to illustrate the dynamic smoothness of the positive coupling between rural public services and rural tourism. It aims to realize resource-sharing, enhance resource utilization efficiency, and promote the coordinated development of both systems.

1. In areas where the level of rural public services is inadequate, such as Yunnan, Guangxi, Guizhou, Chongqing, etc., higher-level governments should increase their financial support to strengthen the financial security of rural public services. Since rural grassroots financial resources are limited, the local government should adopt a multi-party supply mode rather than relying solely on the government. Industrial linkage of public service supply should be introduced, and the rural tourism market can be utilized to bring financial, human, and material support. Incentives tailored to the local characteristics should be developed to encourage tourism enterprises, third-party organizations, and "government-led, enterprise-participation" models to accelerate the integration of rural public services and rural tourism development.
2. The government should establish special funds for rural environmental protection, with a specific focus on enhancing investment in household rubbish disposal. For areas close to towns, the "household collection, village concentration, town treatment" operation mode can be adopted, with household rubbish trucks transporting waste to transfer stations, and pre-treatment by village household rubbish handlers before sending it to collection centers. For remote rural and less populated areas, the "household collection, village concentration, village treatment" model can be implemented to establish village-level waste treatment facilities. An incentive mechanism should be formulated to provide subsidies and rewards to villages actively participating in environmental protection and impact assessment issues, motivating their involvement in safeguarding the environment.
3. Strengthen cooperation between medical and health services and rural tourism. Increased investment should be made to improve medical facilities in rural tourism destinations to ensure timely medical treatment for tourists. Governments should formulate appropriate policies for direct medical settlement in other places and provide accessible medical protection services for tourists and rural residents. The quality of medical services is crucial, so medical service providers should upgrade medical equipment and enhance the professional skills and service level of rural medical personnel. Moreover, exploring and promoting medical services with the characteristics of Southwest China, such as Zhuang medicine, Miao medicine, and Yi medicine, can enhance the attractiveness and competitiveness of rural tourism.

Nevertheless, there are certain restrictions on this study, though. Due to the significant impact of unforeseen events on China’s tourism business, data from 2020 and beyond were excluded from this analysis. Future studies should expand the time frame to acquire more comprehensive results. Furthermore, information technology services represent an important sub-system of public services. However, due to the lack of statistics on this sub-system in rural areas, it has not been included in the indicator system of this paper.

Future studies are encouraged to incorporate more comprehensive and scientifically-based indicators as statistical data are improved and consolidated. Additionally, it should be noted that the development structure and processes of the rural tourism industry vary across different provinces, leading to spatial heterogeneity. This spatial variation must be taken into account when conducting further research and policy planning in the field of rural development and tourism. Therefore, diverse methods, such as spatial effects analysis, can be tried to study the coupling relationship between the two.

## 6. Conclusions

This study measured the development level of the two systems by using the entropy value method and the comprehensive evaluation model, with a focus on southwestern China (study period: 2012–2019). Furthermore, the coupled coordination degree model measured the coupled and coordinated development level of rural public services and rural tourism. By employing the driving factors associated with the coordinated development of the two systems, a single-factor and multi-factor interaction detection analysis was conducted, utilizing Geodetector. Consequently, the following conclusions were obtained:

1. In southwest China, both the rural public service system and the rural tourism system have experienced a fluctuating upward trend in their development. However, the spatial patterns of these two systems revealed uneven results in regional development by the year 2019.
2. Rural public services and rural tourism are interconnected and influence each other, indicating a coupling relationship between the two systems. The degree of linkage coordination between the two systems underwent two stages of "slow increase and accelerated increase" across southwestern China. Chongqing, Yunnan, Guangxi, and Guizhou transitioned from the disordered phase to the intermediate coordination stage, while Sichuan transitioned from the intermediate coordination stage to the advanced stage. Throughout the study period, the type of coupling coordination in all five provinces shifted from rural tourism being less advanced to rural public services being less advanced.
3. The coupling coordination between rural public services and rural tourism is influenced by multiple factors and interactions. The main causative factors, listed in order of influence, are the rural household waste disposal inputs, the number of rural tourism visits, the annual income of rural tourism, and the number of National Leisure Agriculture and Rural Tourism Demonstration Counties (Sites). Regarding interaction detection, the strongest interaction driver is the intersection between the number of health clinics per 10,000 people and the number of China’s Beautiful Leisure Rural. This is followed by the intersection between the number of rural cultural events and the number of China’s Beautiful Leisure Rural, and lastly, the intersection between the annual income of rural tourism and the number of National Leisure Agriculture and Rural Tourism Demonstration Counties (Sites).

## Supporting information

S1 File Origin data of two systems of southwestern China from 2012 to 2019. (ZIP)

S2 File The final calculation results of comprehensive evaluation model. (XLSX)

S3 File The final calculation results of coupling coordination degree model. (XLSX)
